# Structure-Properties Relations for Polyamide 6, Part 2: Influence of Processing Conditions during Injection Moulding on Deformation and Failure Kinetics

**DOI:** 10.3390/polym10070779

**Published:** 2018-07-16

**Authors:** Emanuele Parodi, Gerrit W. M. Peters, Leon E. Govaert

**Affiliations:** 1 Department of Mechanical Engineering, Materials Technology Institute, Eindhoven University of Technology, P.O. Box 513, 5600 MB Eindhoven, The Netherlands; Emanuele.parodi@maxxistce.nl (E.P.); g.w.m.peters@tue.nl (G.W.M.P.); 2 Dutch Polymer Institute (DPI), P.O. Box 902, 5600 AX Eindhoven, The Netherlands

**Keywords:** polyamide 6, injection molding, polymorphism, humidity, mechanical properties

## Abstract

The effect of processing conditions during injection on the structure formation and mechanical properties of injection molded polyamide 6 samples was investigated in detail. A large effect of the mold temperature on the crystallographic properties was observed. Also the the effect of pressure and shear flow was taken in to consideration and analysed. The yield and failure kinetics, including time-to-failure, were studied by performing tensile and creep tests at several test temperatures and relative humidities. As far as mechanical properties are concerned, a strong influence of temperature and relative humidity on the yield stress and time-to-failure was found. A semi-empirical model, able to describe yield and failure kinetics, was applied to the experimental results and related to the crystalline phase present in the sample. In agreement with findings in the literature it is observed that for high mold temperatures the sample morphology is more stable with respect to humidity and temperature than in case of low mold temperatures and this effects could be successfully captured by the model. The samples molded at low temperatures showed, during mechanical testing, a strong evolution of the crystallographic properties when exposed to high testing temperature and high relative humidity, i.e., an increase of crystallinity or a crystal phase transition. This makes a full description of the mechanical behavior rather complicated.

## 1. Introduction

Injection molding is the most widely used technique to produce polymeric products. It is particularly preferred because of advantages such as fast production cycles, cheapness and the large versatility of product shapes. However, injection molding implies some challenges, interesting for users, engineers and polymer scientists. In fact, this technique involves high pressure, elevated shear flow and inhomogeneous transient temperature fields during solidification. The effect of the mold temperature on the mechanical properties is a widely studied topic in polymer engineering. In the case of amorphous polymers, the main influence of mold temperature on the yield stress is attributed to aging; a higher age (i.e., lifetime of a part) of the material correspond to higher yield stress [1]. In the case of semi-crystalline polymers, the topic becomes more complicated. It is well known that injection molded samples do not have a homogeneous morphology along the sample thickness and position [2,3,4]. Ideally, due to different conditions during solidification, three different morphologies can be detected in the sample thickness: (i) the outermost layer (also called skin layer) is subjected to fast cooling, which leads to amorphous or slightly crystalline material; (ii) the central layer (also called core layer) solidifies under high pressure and slow cooling rate, generally leading to high crystallinity; (iii) the inter-phase between the skin and core layer is called shear layer, which is subjected to high shear rates that can play an important role in the crystallization kinetics [5]. Moreover, because of the shear flow, a highly oriented morphology can be formed in this shear layer. The molecular orientation combined with the different position-dependent morphologies creates a strong mechanical anisotropy [4]. The morphology of injection molded nylon 6 was reported in literature [2,6]. These studies led to a common main conclusion: the metastable γ-mesophase (obtainable for moderate cooling rates [7]) is predominant near the surface of the sample, while the most stable α-phase (obtained by slow cooling [7]) takes over towards the center of the sample. The effect of this inhomogeneous morphology distribution on mechanical properties has barely been studied despite this being the most important factor for the end-users. Therefore, in this study, the effect of mold temperature on the mechanical properties of polyamide 6 is investigated. The samples for subsequent mechanical testing were conditioned at different temperatures and different relative humidities.

## 2. State of the Art

In previous studies, the authors have investigated the influence of structural properties (crystalline phase and lamellar thickness), temperature and relative humidity on the yield kinetics and time-to-failure of polyamide 6 processed under quiescent conditions. Firstly, the Ree-Eyring equation, normally used to describe the yield kinetics as a function of temperature and strain rate, was modified in order to include the effect of relative humidity [8]. To accomplish this, the “apparent” temperature equations was introduced:

(1) $$\tilde{T}=T\;+\;\left(T_{g,dry}-T_{g,wet}\right)$$

where *T* is the actual temperature, $T_{g,dry}$ is the glass transition temperature at the dry state and $T_{g,wet}$ is the $T_{g}$ after conditioning. Nex, this “apparent” temperature is substituted in the Ree-Eyring equation:

(2) $$\sigma_{y}\left(\dot{\epsilon },\tilde{T}\right)=\frac{k\tilde{T}}{V_{I}^{*}}\;\sin h^{-1}\left(\frac{\dot{\epsilon }}{{\dot{\epsilon }}_{0,I}}\;\exp\left(\frac{\Delta U_{I}}{R\tilde{T}}\right)\right)+\frac{k\tilde{T}}{V_{II}^{*}}\;\sin h^{-1}\left(\frac{\dot{\epsilon }}{{\dot{\epsilon }}_{0,II}}\;\exp\left(\frac{\Delta U_{II}}{R\tilde{T}}\right)\right)$$

where ${\dot{\epsilon }}_{0,I,II}$, $\Delta U_{I,II}$ and $V_{I,II}^{*}$ are the rate factor, the activation energy and the activation volume related to process I and process II, respectively. These processes are related to two different deformation mechanisms; process I to intra-lamellar deformation and process II to inter-lamellar deformation. Subsequently, this work focused on applying the model also in case of different crystal phases with varying lamellar thicknesses ($l_{c}$). For the two different polymorphs, two sets of parameters were defined, one for α-phase and one for γ-mesophase. Remarkably, the first process of both the polymorphs could be described with identical activation energy ($\Delta U_{I}$) and activation volume ($V_{I}^{*}$) [9]. Also $l_{c}$, a relation between the lamellar thickness and the rate factors was found [9]. Thus, by selecting the right set of parameters accordingly to the present crystal phase, the yield kinetics of different samples with different $l_{c}$ can be described. The parameters defined for α-phase and γ-mesophase are listed in Table 1 and Table 2, respectively.

Moreover, by the use of the “critical strain” concept, the prediction made for the yield kinetics can be reconverted to predict for the time-to-failure [10]. The time-to-failure ($t_{f}$) is estimated by the equation:

(3) $$t_{f}\left(\sigma ,T\right)=\frac{\epsilon_{cr}}{{\dot{\epsilon }}_{pl}\left(\sigma ,T\right)}$$

in which $\epsilon_{cr}$ is the critical strain and $\epsilon_{pl}$ is the plastic flow rate as function of load and temperature obtained by the yield kinetics, see Equation (2).

## 3. Experimental

### 3.1. Material

The material employed in this work was a polyamide 6 (Akulon K122) kindly provided by DSM (Geleen, The Netherlands). This PA6 has a viscosity-average molar mass (Mv) of about 24.9 kg/mol, melting point 220 ${}^{\circ }$C, density of the amorphous phase 1090 ${\mathrm{kg}/m}^{3}$ and a viscosity number of 0.312 $m^{3}$/kg (ISO 307) [11].

### 3.2. Sample Preparation

**Figure 1:**
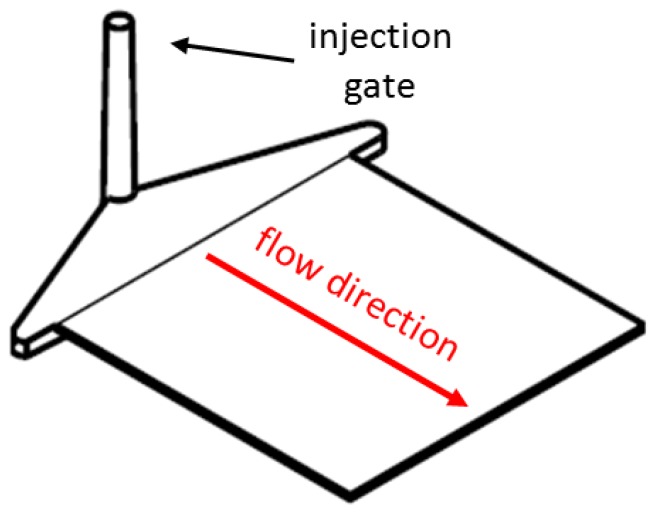
Schematic representation of a sample obtained by injection molding (70 × 70 × 1 mm).

To dry the pellets prior to processing, the material was placed in a vacuum oven at a temperature of 110 ${}^{\circ }$C for 12 h. Next, the injection molding procedure was performed with the following parameters: temperature profile from the hopper to the nozzle, 70 ${}^{\circ }$C, 230 ${}^{\circ }$C, 240 ${}^{\circ }$C, 250 ${}^{\circ }$C, 245 ${}^{\circ }$C and 240 ${}^{\circ }$C; injection flow, 90 cm${}^{3}$/s; maximum injection pressure, 500 bar; holding pressure, 500 bar; cooling time 30 s. Moreover, four mold temperature were used, such as 35, 85, 130 and 160 ${}^{\circ }$C. The samples are 1 mm thick squared plates with side lengths of 70 mm. The injection gate was situated orthogonally to the plate plane, see Figure 1. Dog-bone shape samples were cut by the mean of a cutting die, according to the ISO527 type 1BA (main dimensions: width 5 mm, length 22 mm). The samples were cut in parallel and perpendicular direction compared to the flow, see Figure 1.

As supporting experiments, also sheets with a thickness of 0.5 mm were prepared by compression molding (for a more extensive study on the effects of injection molding see [9]). The material was melted at 265 ${}^{\circ }$C for 5 min, while a force of about 10 kN was applied, then it was rapidly moved to a cold press set at different temperatures, i.e., 80–120–140–160–180 ${}^{\circ }$C where the material was solidified for 3 min.

### 3.3. Sample Conditioning

The samples were conditioned at room temperature (23 ${}^{\circ }$C) at four different relative humidities: RH 0% (dry) using a vacuum chamber at room temperature, RH 35% and RH 75% by using chambers containing supersaturated solutions of sodium chloride and magnesium chloride hexahydrate, and RH 50% by using an environmental chamber. The samples were kept in the conditioning environment up to saturation. The absorbed water fraction was calculated with the following equation:

(4) $$H_{2}O\%=\left(\frac{W_{i}-W_{0}}{W_{0}}\right)\times 100$$

where $W_{0}$ is the weight of the sample in dry conditions and $W_{i}$ is the weight at the time $t_{i}$. The saturation was identified as the level of water fraction after which a plateau is reached. Since water is mainly absorbed in the amorphous phase we define the normalized water fraction in which the water fraction is related to the the fraction of amorphous phase:

(5) $$H_{2}O{\%}_{N}=\frac{H_{2}O\%}{1-\chi_{vol}}$$

where $\chi_{vol}$ is the crystallinity.

### 3.4. Mechanical Tests

To investigate the yield and failure kinetics, uniaxial tensile and creep tests were performed using a Zwick Uniaxial Testing Machine (Ulm, Germany) equipped with a 1 kN load-cell and an environmental chamber with which temperature and relative humidity were controlled. Relative humidity ranged from RH 35% to RH 75%, strain rates from $10^{-5}$ $s^{-1}$ up to $3\times 10^{-2}$ $s^{-1}$ and temperatures from 23 to 120 ${}^{\circ }$C. Each experiment was performed at least two times. As far as the the tensile tests is concerned, a pre-load of 0.1 MPa was applied prior the test with a speed of 1 mm/min. Subsequently the test was performed with constant strain rate up to a strain of ≈50%. The creep tests were performed at room temperature (23 ${}^{\circ }$C) and three relative humidities (RH 35%, RH 50% and RH 75%). The load was applied within 10 s, then it was kept constant up to failure. Because of analytical issues, the time-to-failure was defined as the time to reach a strain of 35%.

### 3.5. X-ray Diffraction

To investigate the influence of different mold temperatures on structure, X-ray diffraction experiments were performed. First, wide angle X-ray diffraction (WAXD) measurements were done using a Ganesha X-ray instrument (Copenhagen, Denmark) equipped with a GeniX-Cu ultra low divergence source (l = 1.54 Å) and a Pilatus 300 K silicon pixel detector (487 × 619 pixels of 172 × 172 $\mu m^{2}$). The patterns obtained were radially integrated and the weight percentage crystallinity was estimated by:

(6) $$\chi_{c}=\frac{T-A}{T}$$

where *T* is the total scattered intensity and *A* is the scattering from the amorphous halo. The amorphous halo was retrieved performing WAXD on a completely amorphous sample obtained by quenching the material in water with ice and NaCl. However, especially in the case of injection molding, the material can crystallize in a mixture of α and γ. Thus the total crystallinity calculated by Equation (6) may actually consist of a fraction related to the α-phase and another fraction related to γ-mesophase. Therefore, a deconvolution analysis is performed. It consist of an analytical fitting of Lorentzian’s functions to the characteristics peaks. Next, all the function areas are summed up to the amorphous halo area, and the resulting pattern is compared to the experimental result. The α-phase and γ-mesophase fractions follow from:

(7) $$\chi_{c,\alpha }=\frac{A_{\alpha }}{A_{m}}\;\;\;\;\;\;\;and\;\;\;\;\;\chi_{c,\gamma }=\frac{A_{\gamma }}{A_{m}}$$

where $A_{\alpha }$ and $A_{\gamma }$ are the total area of the Lorentzian functions for the α and γ peaks, and $A_{m}$ is the total area of the measured pattern. Also, small angle X-ray scattering (SAXS) was performed on the same setup described for WAXD, only the samples to detector distance was increased. With SAXS, the difference of electronic density are detected, i.e., the distance covered by a lamella and an amorphous layer. This is called the long period ($L_{b}$). After applying Lorentz [12] and thermal density fluctuation [13] corrections, the long period is calculated with:

(8) $$L_{b}=\frac{2\pi }{d^{*}}$$

where $d^{*}$ is the peak position expressed in inversed nanometers (${\mathrm{nm}}^{-1}$). Next, the lamellar thickness ($l_{c}$) is estimated by:

(9) $$l_{c}=\chi_{vol}\cdot l_{b}$$

where $\chi_{vol}$ is the volumetric crystallinity percentage, which takes into account the different density of amorphous phase and the two polymorphs (α and γ):

(10) $$\chi_{vol}=\frac{\frac{\chi_{c}}{\rho_{c}}}{\frac{\chi_{c}}{\rho_{c}}+\frac{100-\chi }{\rho_{a}}}$$

where $\rho_{c}$ is the density of the crystal (1.21 g/cm${}^{3}$ for α-phase [11], 1.16 g/cm${}^{3}$ for γ-mesophase [11]), $\rho_{a}$ the density of the amorphous (1.09 g/cm${}^{3}$[11]) and χ the mass crystallinity. To investigate the influence of shear flow during crystallization, an azimuthal integration (180°) of the SAXS patterns was performed.

### 3.6. Dynamical Mechanical Thermal Analysis

To investigate the influence of processing and relative humidity on the glass transition temperature, a TA instruments Q800 (Asse, Belgium) was used to perform dynamical mechanical thermal analysis (DMTA). Flat rectangular samples (main sizes 0.5 × 5 mm) were tested performing a ramp of temperature from −40 ${}^{\circ }$C to 120 ${}^{\circ }$C with a heating speed of 3 ${}^{\circ }$C/min and a frequency of 1 Hz. The glass transition temperature was defined ad the peak of the tan(δ) curve.

### 3.7. Dilatometry-PVT

A dilatometer (PVT) able to measure the specific volume of polymers as a function of cooling rate, shear flow, pressure and temperature, was employed [14]. It consists of a pressure cell which combines a traditional “piston-die” type dilatometer with a Couette geometry rheometer. The experiments were performed on ring-shaped samples produced by a mini injection molding machine (Babyplast, Molteno, Italy), with main dimensions: 22 mm outer diameter, 21 mm inner diameter and height of of 2.5 mm. To completely erase the thermal history, the sample was heated at 250° and kept at this temperature for 10 min. Then, the cooling procedure was performed for isobaric conditions. Two kind of cooling procedures were applied, ambient cooling (≈0.1 ${}^{\circ }$C/s) and air cooling (≈1 ${}^{\circ }$C/s). The pressure was varied in a range from 100 bar to 800 bar and the shear flow in range from 0 s${}^{-1}$ to 180 s${}^{-1}$. The influence of shear flow was studied at 200 bar and the shear impulse was applied at 200 ${}^{\circ }$C.

## 4. Results and Discussion

### 4.1. Samples Characterization

**Figure 2:**
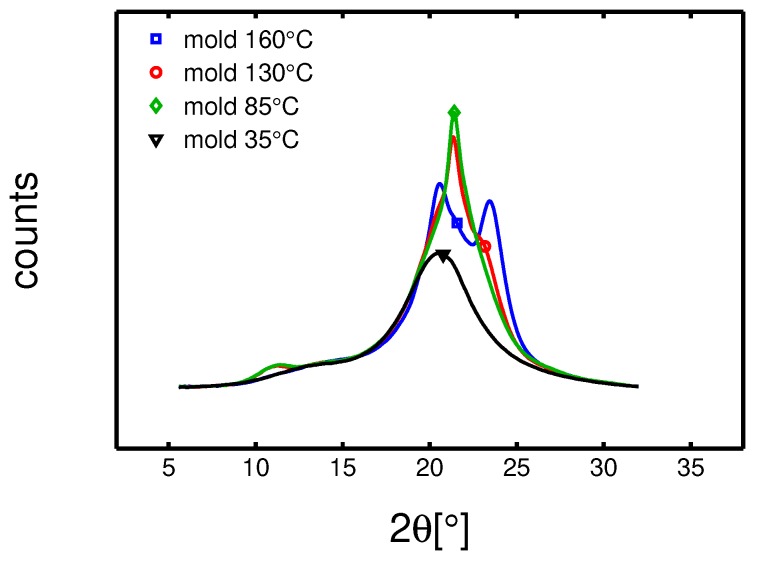
WAXD integrated patterns of samples molded at different temperatures; experiments performed at room temperature (23 ${}^{\circ }$C) and dry condition.

**Figure 3:**
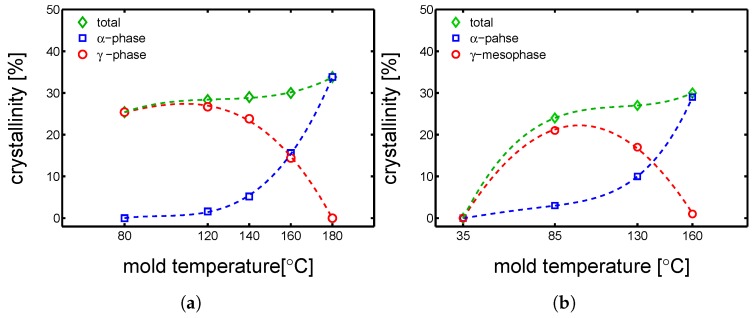
Deconvolution analysis of WAXD patterns. Crystallinity, α and γ fractions as functions of mold temperature. (**a**) Case: compression molding, (**b**) case: injection molding.

The first step of this study was a crystallographic characterization performed by WAXD and SAXS experiments (details about these techniques are reported in Section 3.5). WAXD experiments were performed on dry samples to understand the influence of mold temperature. In Figure 2, the integrated patterns are reported. It is observed that the mold temperature plays a crucial role on the crystallization: (i) at 35 ${}^{\circ }$C an almost completely amorphous sample is obtained, (ii) at 85 ${}^{\circ }$C a predominantly γ-mesophase sample is obtained, (iii) at 130 ${}^{\circ }$C a mixture of α-phase and γ-mesophase are found and (iiii) at 160 ${}^{\circ }$C a fully α-phase sample is obtained. In Figure 3b, the result of the deconvolution analysis is shown.

It is important to stress that, the results of X-ray analysis are all an average over the thickness. Normally, the cross section of an injection molded part shows a not homogeneous morphology. During processing, the material is exposed to very different conditions dependent on the history experienced in the mold. The material close to mold surface solidifies upon high cooling rate (generally called skin layer), the middle part solidifies relatively slowly under high pressure (called core layer), while the layer between the core and skin layer, solidifies under high shear (it is called shear layer). However, because of experimental limitations, it was chosen to present the results as an average over the thickness. In Figure 3a, the deconvolution analysis of samples compression molded at different temperatures are presented. Comparing the results shown in Figure 3a,b it possible to notice that in the case of injection molding, already a substantial fraction of α-phase is formed at a mold temperature of 130 ${}^{\circ }$C, while in the case of compression molding mold temperature 130 ${}^{\circ }$C leads to a sample with a rather small fraction of α-phase and a predominance of γ-mesophase. In order to study this effect, we have performed supporting PVT experiments.

#### 4.1.1. Supporting Experiments-PVT

**Figure 4:**
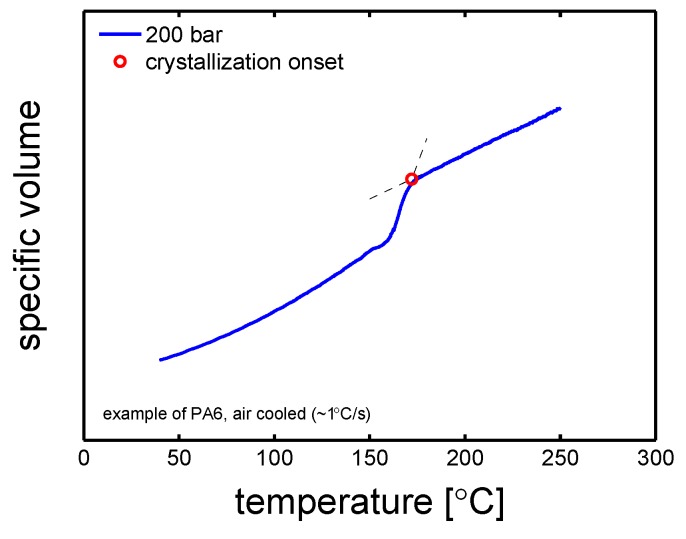
Dilatometry-PVT experiment, cooling at ≈1 ${}^{\circ }$C/s and 200 bar with no shear flow. The circle indicates the crystallization onset temperature.

**Figure 5:**
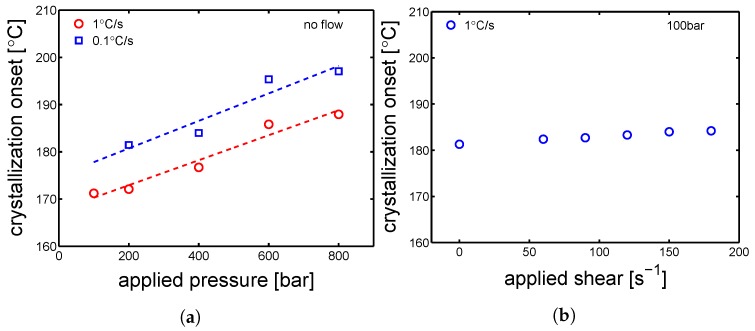
(**a**). Crystallization onset temperature obtained by cooling upon different pressures and cooling rates. Lines are just guides to the eye. (**b**) Crystallization onset temperature obtained by cooling upon different shear flow rates and 100 bar.

A dilatometer (PVT) was employed in order to perform cooling procedures that simulated processing conditions, i.e., crystallization during cooling at high pressures and subjected to shear flows. In Figure 4 an illustrative example of a PVT result is presented. The sample was cooled at ≈1 ${}^{\circ }$C/s, with a constant pressure of 200 bar and no shear. The marker (circle) at about 172 ${}^{\circ }$C (see Figure 4) is defined as the crystallization onset, which is the main outcome of this experiment. As far as the influence of pressure on the crystallization onset temperature is concerned, cooling at two different speeds (0.1 and 1 ${}^{\circ }$C/s) were performed for several pressures (from 100 to 800 bar). The crystallization onset for these conditions is plotted as a function of applied pressures, see Figure 5a. The results show an increase of crystallization onset of about 17 ${}^{\circ }$C from the minimum to the maximum applied pressure in both the investigated cooling rates. In the case of crystallization with applied shear flow and constant pressure (100 bar), the crystallization onset increases only few degrees. Therefore, it is concluded that the different crystallization behavior observed for the injection molding processing, compared to the one seen for compression molding, is mainly due to the effect of pressure which increases the crystallization temperature for a given cooling rate.

#### 4.1.2. Effect of Flow on the Molecular Orientation

**Figure 6:**
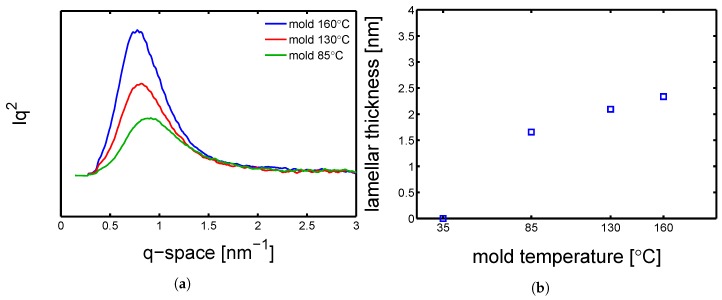
(**a**) SAXS integrated patterns of samples molded at different temperatures; experiments performed at room temperature (23 ${}^{\circ }$C) and dry condition. (**b**) Lamellar thickness as a function of mold temperature; experiments performed at room temperature (23 ${}^{\circ }$C) and dry condition.

Next, SAXS experiments were performed on the dry samples processed at different mold temperatures. In Figure 6a, the results of radial integration of the SAXS patterns, which give the required information for to estimating the average long period ($l_{b}$) and, as explained in Section 3.5, the average lamellar thickness ($l_{c}$). Figure 6b shows the lamellar thickness values as functions of mold temperature; the highest $l_{c}$ (≈2.3 nm) is obtained with mold temperature 160 ${}^{\circ }$C and the minimum (≈1.6 nm) at mold temperature 85 ${}^{\circ }$C.

**Figure 7:**
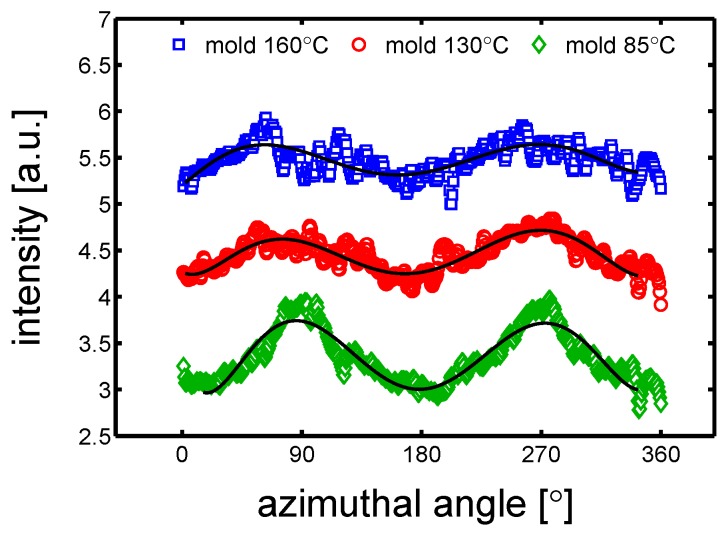
Azimuthal integration over a range from 0° to 180° of samples at dry conditions and room temperature. The solid lines are guide to the eye.

As mentioned in the introduction, flow may lead to material orientation and this could affect the mechanical properties. To investigate the whether or not orientation was present, an azimuthal integration of the SAXS pattern was performed. In Figure 7 three examples of azimuthal integration of samples produced at different mold temperatures and conditioned at RH 0% (dry) at room temperature, are shown. A varying orientation is observed for all the three samples; in particular the mold temperature 85 ${}^{\circ }$C shows two clear maximums at approximately 90° and 270°.

**Figure 8:**
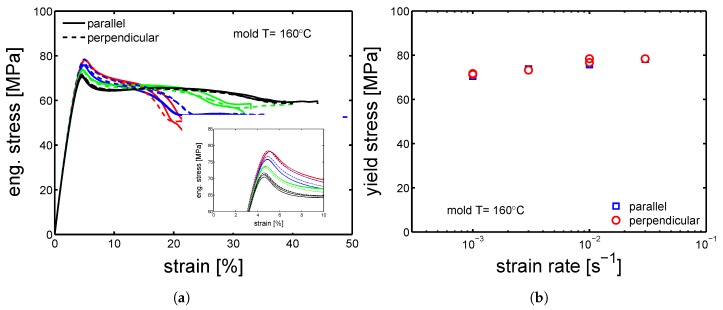
(**a**) Tensile tests at strain rate from $10^{-3}$ s${}^{-1}$ to 3× $10^{-2}$ s${}^{-1}$ at 23 ${}^{\circ }$C; comparison between samples cut in parallel (solid lines) and perpendicular (dashed lines) direction compare to the flow. (**b**) Yield stress as a function of strain rate. Samples molded at 160 ${}^{\circ }$C.

**Figure 9:**
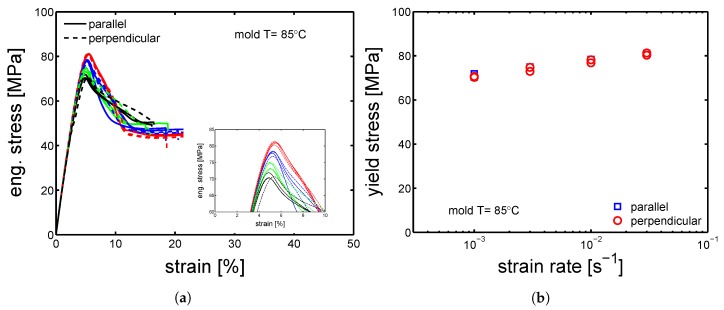
(**a**) Tensile tests at strain rate from $10^{-3}$ s${}^{-1}$ to 3× $10^{-2}$ s${}^{-1}$ and 23 ${}^{\circ }$C; comparison between samples cut in parallel (solid lines) and perpendicular (dashed lines) direction compare to the flow. (**b**) Yield stress as a function of strain rate. Samples molded at 85 ${}^{\circ }$C.

Therefore, the mechanical properties of samples cut in parallel and perpendicular direction compare to the flow direction, were tested by tensile tests at different strain rates. The stress-strain response of “parallel” and “perpendicular” sample molded at 160 ${}^{\circ }$C are presented in Figure 8a. Astonishingly, both the “parallel” and “perpendicular” samples showed the same yield stress (±1 MPa), see Figure 8b. The same observations were made for the samples molded at 85 ${}^{\circ }$C, see Figure 9a,b.

### 4.2. Yield Kinetics

**Figure 10:**
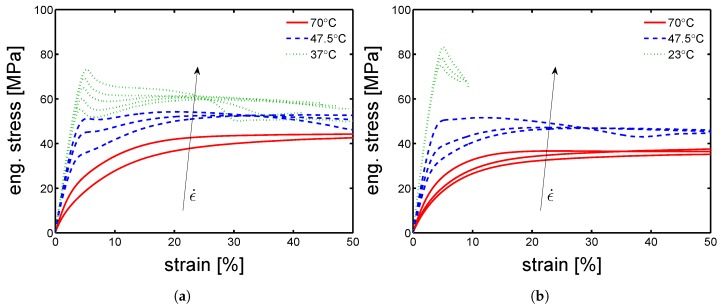
Stress-strain response at different temperatures and strain rates of samples molded at (**a**) 160 ${}^{\circ }$C and (**b**) 130 ${}^{\circ }$C.

**Figure 11:**
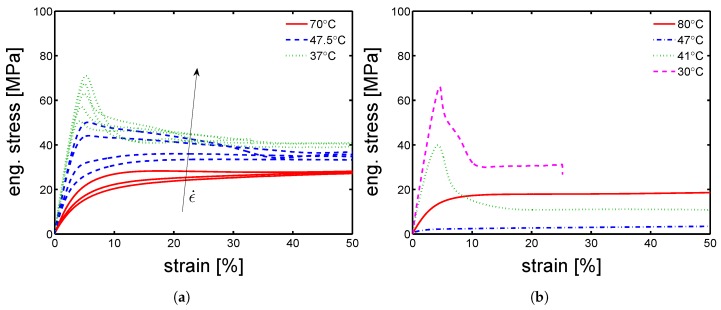
Stress-strain response at different temperatures and strain rates of samples molded at (**a**) 85 ${}^{\circ }$C and (**b**) 35 ${}^{\circ }$C.

Because of the absence of effect of the orientation on the mechanical properties, the study continued on “parallel” samples only. The mechanical characterization continued with the investigation of yield kinetics for dry conditions. Uniaxial tensile test were performed in a range of temperatures from 23 ${}^{\circ }$C to 80 ${}^{\circ }$C and strain rates from $3\times 10^{-4}$ up to $3\times 10^{-2}$ s${}^{-1}$. In Figure 10a,b and Figure 11a,b, examples of stress-strain response at different temperatures are presented for mold temperature of 160 ${}^{\circ }$C, 130 ${}^{\circ }$C, 85 ${}^{\circ }$C and 35 ${}^{\circ }$C respectively. As expected, the yield stress increases for increasing strain rates and decreases for increasing temperature. However, this statement does not hold for the samples molded at 35 ${}^{\circ }$C. For these, the increase of temperature from 23 ${}^{\circ }$C to 47.5 ${}^{\circ }$C leads rapidly to a dramatic drop of yield stress (from ≈70 MPa to ≈5 MPa), while increasing even further the temperature, the measured yield stress rises up to ≈20 MPa, see Figure 11b. This is a clear indication of the evolution of the sample state. Indeed, the samples molded at 35 ${}^{\circ }$C are almost completely amorphous samples, see Figure 2. Heating an amorphous samples above its $T_{g}$ causes cold crystallization and, consequently an enhancement of the yield stress is obtained. This effect is also visible in the DMTA experiments which are presented later on (see Figure 15a). The results for a mold temperature of 160 ${}^{\circ }$C showed the weakest dependence on testing temperature and strain rate, while 85 ${}^{\circ }$C showed the strongest (apart from the mold temperature 35 ${}^{\circ }$C, which is a different case).

**Figure 12:**
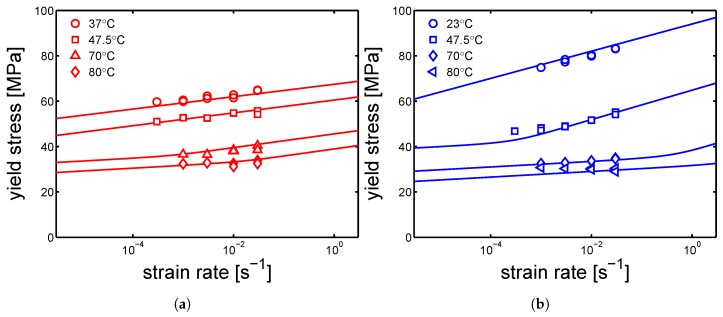
Yield kinetics (yield stress as a function of strain rate) of samples in dry condition, molded at (**a**) 160 ${}^{\circ }$C (**b**) 130 ${}^{\circ }$C. Lines are the the results of the Ree-Eyring equation.

**Figure 13:**
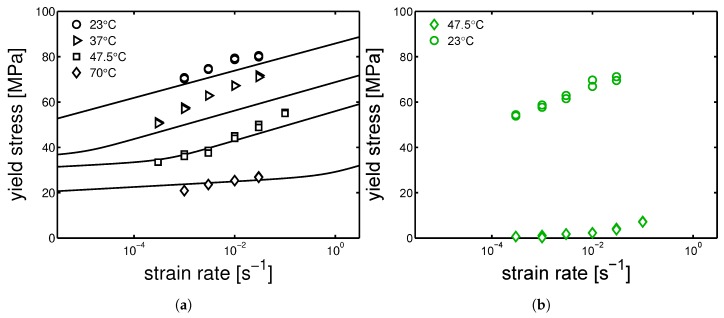
Yield kinetics (yield stress as a function of strain rate) of samples in dry condition, molded at (**a**) 85 ${}^{\circ }$C (**b**) 35 ${}^{\circ }$C. Lines are the the results of the Ree-Eyring equation.

In Figure 12a,b and Figure 13a,b, the yield kinetics (yield stress as a function of strain rate) of dry samples are given. For the cases of samples molded at 160 ${}^{\circ }$C and 130 ${}^{\circ }$C, in which α-phase (160 ${}^{\circ }$C) and γ-mesophase (130 ${}^{\circ }$C) are predominant, predictions based on Equation (2) (in which $\tilde{T}$ is just the testing temperature) using parameters listed in Table 1 for mold 160 ${}^{\circ }$C and Table 2 for 130 ${}^{\circ }$C are given by the lines in Figure 12a,b. As explained in Section 2, the rate factors are defined accordingly to the lamellar thickness, see Table 3, Table 4 and Table 5. The lines match the experimental results rather well for these two cases. However, for a mold temperature of 85 ${}^{\circ }$C, a clear mismatch between the prediction and the experimental results is found, see Figure 13a. In this case, the parameters related to γ-mesophase are employed. However, it is evident that the strain rate and temperature dependence of the yield stress is stronger then in the case of a mold temperature of 130 ${}^{\circ }$C and 160 ${}^{\circ }$C. This difference is even more clear for the results obtained at 37 ${}^{\circ }$C, see Figure 13a. Figure 11b shows the stress-strain response of samples molded at 35 ${}^{\circ }$C; the results obtained in this case are far from what found for a mold temperatures of 130 ${}^{\circ }$C and 160 ${}^{\circ }$C.

**Figure 14:**
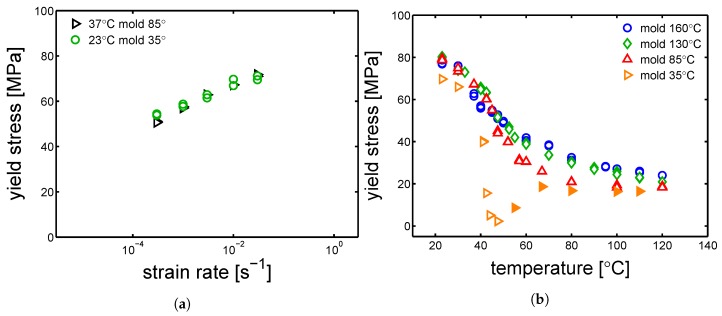
(**a**) Comparison between the yield kinetics of samples molded at 85 ${}^{\circ }$C and 35 ${}^{\circ }$C. (**b**) Yield stress as a function of temperature. In the case of the samples molded at 35 ${}^{\circ }$C, the transition from unfilled to filled markers, is due to the fact that after ≈45 ${}^{\circ }$C the samples start to cold-crystallize. Thus, the filled markers are not really representative of the samples molded at 35 ${}^{\circ }$C but an evolution of those. Strain rate $10^{-2}$ s${}^{-1}$.

However, comparing results for a given mold temperature of 85 ${}^{\circ }$C (Figure 13a) and a mold temperature 35 ${}^{\circ }$C (Figure 13b), a very similar strain rate dependence (slope) of the yield stress is found at low temperatures, see Figure 14a. The reason of this behavior might be found by considering the samples homogeneity. As previously explained, injection molding samples show a non-homogeneous morphology along the thickness. It is likely that, in the case of mold temperature 85 ${}^{\circ }$C, the skin layer (i.e., the outermost layer) is thicker than in the case of 130 ${}^{\circ }$C and 160 ${}^{\circ }$C, and thus a predominant contribution of the skin layer leads to a behavior closer to an amorphous sample rather then a semi-crystalline.

In Figure 14b, the yield stress obtained by tensile test at strain rate $10^{-2}$ s${}^{-1}$ is plotted as a function of temperature in a range from 23 ${}^{\circ }$C to 120 ${}^{\circ }$C. This figure shows clearly the behavior of the samples molded at 35 ${}^{\circ }$C when tested at higher temperatures: at temperatures lower than ≈40 ${}^{\circ }$C yield stress is slightly lower than the other samples molded at higher temperatures. Above ≈40 ${}^{\circ }$C the yield stress decreases rapidly down to a minimum of about 5 MPa at 47.5 ${}^{\circ }$C; increasing the temperature further, the yield stress increases again and at about 80 ${}^{\circ }$C it reaches a plateau that continues up to the highest temperature investigated (120 ${}^{\circ }$C). The samples molded at 130 ${}^{\circ }$C and 160 ${}^{\circ }$C show a similar behavior if plotted as function of temperature. The absolute value is slightly higher in the case of a mold temperature 160 ${}^{\circ }$C and 130 ${}^{\circ }$C. This is due to a larger lamellar thickness. At testing temperatures between 23 ${}^{\circ }$C and ≈45 ${}^{\circ }$C, the samples molded at 85 ${}^{\circ }$C show an yield stress comparable with mold 130 ${}^{\circ }$C and 160 ${}^{\circ }$C; above ≈45 ${}^{\circ }$C, the yield stress drops moderately till a minimum at is reached 120 ${}^{\circ }$C, see Figure 14b.

### 4.3. Influence of the Conditioning Environment

**Figure 15:**
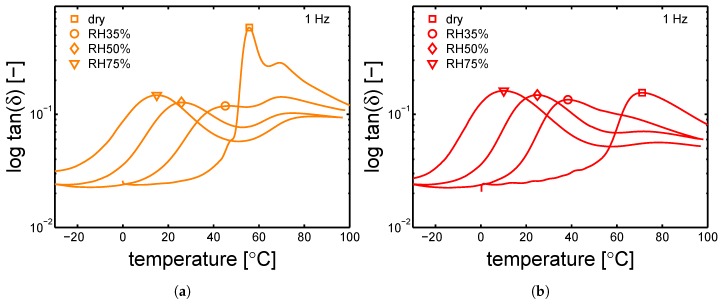
DMTA experiments for samples conditioned at different relative humidities, tan(δ) as a function temperature for samples molded at (**a**) 35 ${}^{\circ }$C and (**b**) 85 ${}^{\circ }$C. Markers indicate the measured $T_{g}$’s.

**Figure 16:**
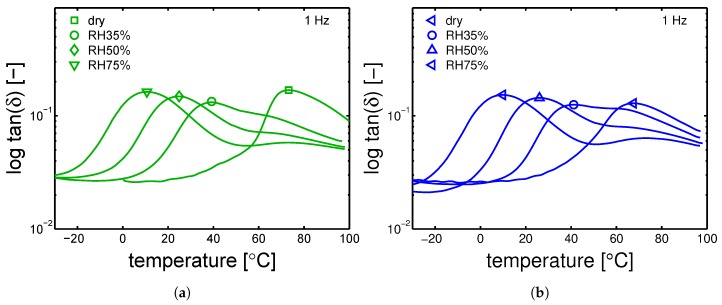
DMTA experiments for samples conditioned at different relative humidities, tan(δ) as a function temperature for samples molded at (**a**) 130 ${}^{\circ }$C and (**b**) 160 ${}^{\circ }$C. Markers are the selected $T_{g}$.

The glass transition temperatures were measured by DMTA, after conditioning at a relative humidity 35%, 50% and 75% at room temperature (23 ${}^{\circ }$C). The results are shown in Figure 15a,b and Figure 16a,b. The $T_{g}$ values are reported in Table 6. In Figure 15a, the cold crystallization previously mentioned, is clearly visible by observing the curve related to the dry sample at ≈60 ${}^{\circ }$C.

The DMTA results for the samples that are conditioned at different humidities are vital in order to determine the “apparent” temperature (Equation (1)), see Section 2. The markers indicate the estimated glass transition temperatures.

**Figure 17:**
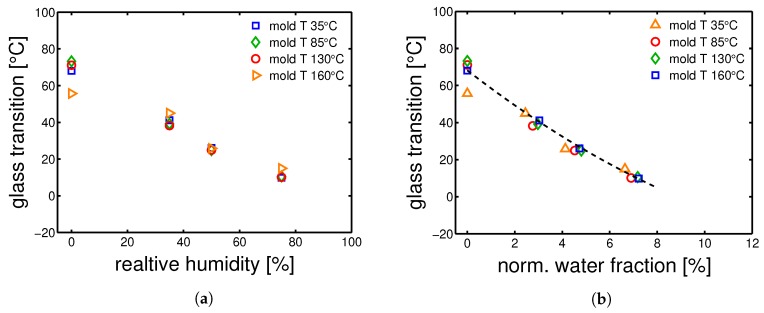
Glass transition temperatures as functions of (**a**) relative humidities and (**b**) normalized absorbed water fraction. The line is a guide to the eye.

The glass transition temperatures are given as a function of the relative humidity (during conditioning), see Figure 17a. The largest differences in glass transition temperature are found in the case of a mold temperature 35 ${}^{\circ }$C and dry condition. After conditioning, all the samples show a very similar $T_{g}$. The measured $T_{g}$ are reported in Table 6. Finally, the glass transition temperatures can be also plotted as functions of normalized water fraction (see Equation (5)), which takes into account that only the amorphous fraction can absorb water [15]. Figure 17b shows that, when plotting $T_{g}$ as a function of normalized water fraction, all the results are captured by a monotonic descending trend line.

#### 4.3.1. Hydration-Induced Crystallographic Evolution

**Figure 18:**
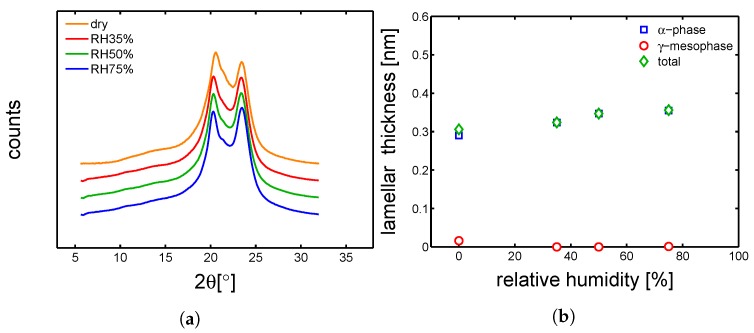
(**a**) Wide angle X-ray diffraction integrated patterns, samples molded at 160 ${}^{\circ }$C and conditioned at different humidities. (**b**) Deconvolution analysis of the integrated WAXD patterns, crystalline, α and γ fraction as a function of relative humidity.

**Figure 19:**
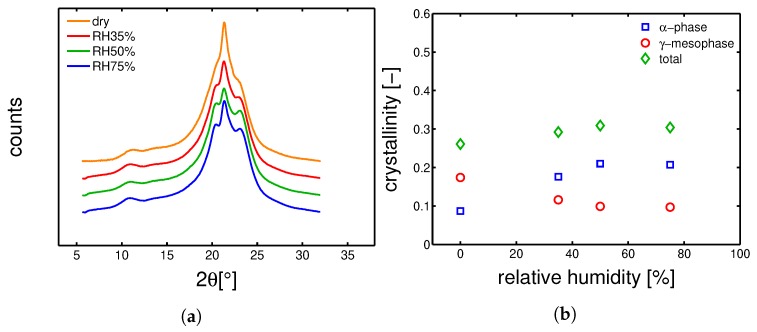
(**a**) Wide angle X-ray diffraction integrated patterns, samples molded at 130 ${}^{\circ }$C and conditioned at different humidities. (**b**) Deconvolution analysis of the integrated WAXD patterns, crystalline, α and γ fraction as a function of relative humidity.

**Figure 20:**
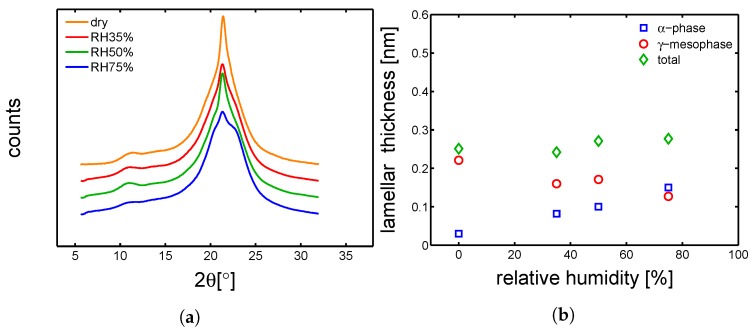
(**a**) Wide angle X-ray diffraction integrated patterns, samples molded at 85 ${}^{\circ }$C and conditioned at different humidities. (**b**) Deconvolution analysis of the integrated WAXD patterns, crystalline, α and γ fraction as a function of relative humidity.

**Figure 21:**
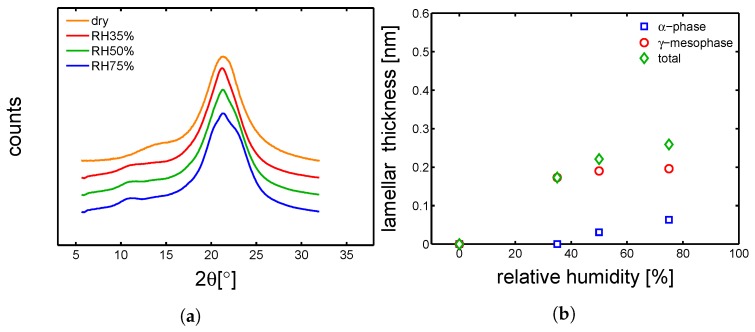
(**a**) Wide angle X-ray diffraction integrated patterns, samples molded at 35 ${}^{\circ }$C and conditioned at different humidities. (**b**) Deconvolution analysis of the integrated WAXD patterns, crystalline, α and γ fraction as a function relative humidity.

In order to check the influence of hydration on the crystallographic properties, WAXD experiments were carried out on conditioned samples. In Figure 18a, Figure 19a, Figure 20a and Figure 21a, the integrated patterns are shown for the case of mold 160 ${}^{\circ }$C, 130 ${}^{\circ }$C, 85 ${}^{\circ }$C and 35 ${}^{\circ }$C; the corresponding deconvolution analysis of these patterns are given in figure Figure 18b, Figure 19b, Figure 20b and Figure 21b. The drop of glass transition, due to water absorption, has different effects depending on the starting morphology. In the case of a mold temperature of 160 ${}^{\circ }$C only a slight increase of crystallinity is recorded, most probably due to secondary crystallization. The samples molded at 130 ${}^{\circ }$C show a partial transformation from γ to α-phase and a slight increase of the overall crystallinity. Similar behavior is seen in the case of mold temperature 85 ${}^{\circ }$C. Finally, in the case of 35 ${}^{\circ }$C, the cold crystallization leads, initially, to the crystallization of γ-mesophase (at RH 35%) and at higher relative humidity also α-phase is crystallized.

#### 4.3.2. Effect of Water Absorption on the Mechanical Response

**Figure 22:**
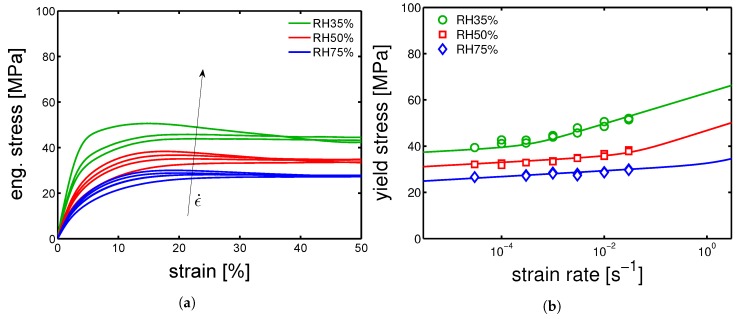
(**a**) Stress-strain response of samples conditioned at different relative humidities and tested at strain rate in a range from 3× $10^{-5}$ s${}^{-1}$ to 3× $10^{-2}$ s${}^{-1}$. (**b**) Yield stress kinetics of samples molded at 130 ${}^{\circ }$C.

**Figure 23:**
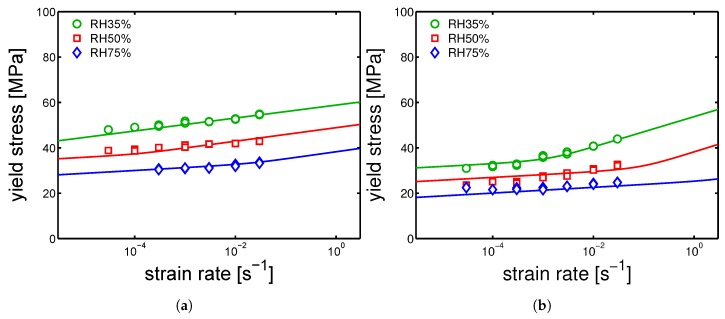
Yield stress kinetics of samples conditioned at 23 ${}^{\circ }$C different relative humidities and molded at (**a**) 160 ${}^{\circ }$C and (**b**) 85 ${}^{\circ }$C.

After conditioning and determination of the glass transition temperature, uniaxial tensile tests were performed in environments with controlled temperature and relative humidity. The temperature was kept constant at 23 ${}^{\circ }$C and three relative humidities were selected, RH 35%, RH 50% and RH 75%. In Figure 22a, an example of the effect of humidity on the stress-strain response of samples molded at 130 ${}^{\circ }$C is presented. As already mentioned, the increase of relative humidity leads to a decrease of mechanical response. The yield stress values obtained at different relative humidity are plotted as function of the applied strain rate, see Figure 22b. The symbols are the values obtained experimentally, whereas the lines are the predictions based on the Equation (2) in which, in order to include the influence of RH% on the mechanical properties, the temperature was replaced by the “apparent” temperature equation. The agreement is excellent. The yield kinetics for samples molded at 160 ${}^{\circ }$C and 85 ${}^{\circ }$C are shown in Figure 23a,b, respectively. As in the case of dry samples, the predictions made for samples molded at 160 ${}^{\circ }$C and 130 ${}^{\circ }$C match quite well the experimental results. Even for the 85 ${}^{\circ }$C mold temperature case, for which the agreement between model and experiment was not satisfactory for dry condition the description (lines) are not too far from the experimental results. Our explanation for this results is related to the effect of hydration on the crystallographic properties. As explained previously, because of the drop of glass transition due to hydration, cold crystallization is observed in the case of amorphous (quenched) samples. Thus, the amorphous skin layer of the samples molded at 85 ${}^{\circ }$C are likely to cold-crystallize in γ-mesophase, see Figure 21b. Consequently, a decreased amorphous contribution on the mechanical properties is obtained and by the use of the parameters for γ-phase, a rather good description is obtained. Moreover, the hydration-induced drop of $T_{g}$ decreases the mechanical contribution of the amorphous regions.

### 4.4. Creep Tests

**Figure 24:**
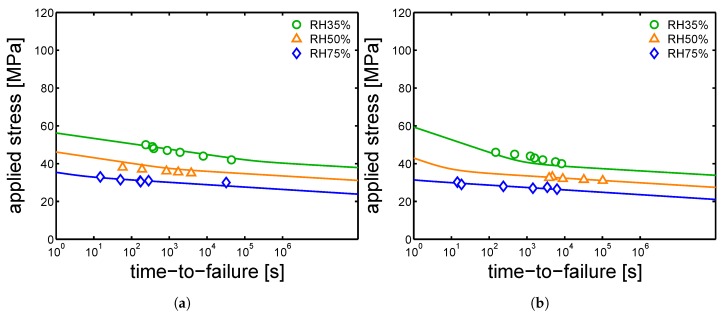
Creep tests, applied load as a function of time-to-failure for samples conditioned at different relative humidities and room temperature. The lines are the results of Equation (3). (**a**) Mold temperature 160 ${}^{\circ }$C and (**b**) 130 ${}^{\circ }$C.

**Figure 25:**
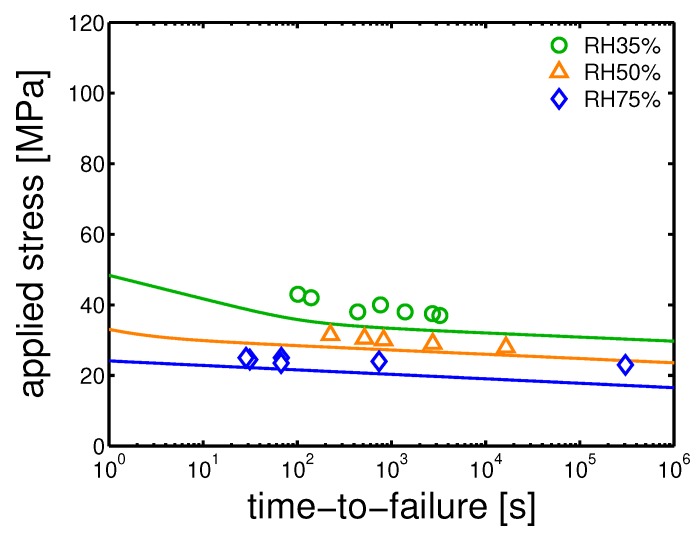
Creep tests, applied load as a function of time-to-failure for samples conditioned at different relative humidities and room temperature. The lines are the results of Equation (3). Mold temperature 85 ${}^{\circ }$C.

Finally, creep test at different relative humidity were performed. The samples were tested at 23 ${}^{\circ }$C and different loads were applied. The time-to-failure (t-t-f) was defined as the time to reach a strain of 35%, and the plastic flow rate is calculated by selecting the minimum in the Sherby-Dorn plot [16]. Subsequently, from the plastic flow rates, plotted as functions of the corresponding measured time-to-failure in a bi-logarithmic plot, the critical strain ($\epsilon_{cr}$) is determined, as explained in [10]. Next, using the predictions made for the yield kinetics, Equation (3) is applied. In Figure 24a,b and Figure 25, the results of creep tests at different relative humidity and several applied load are shown for the samples injection molded at 160 ${}^{\circ }$C, 130 ${}^{\circ }$C and 85 ${}^{\circ }$C, respectively.

The experimental results for mold temperatures of 160 ${}^{\circ }$C and 130 ${}^{\circ }$C are well described by the model (see Figure 24a,b). However, in the case of a mold temperature of 85 ${}^{\circ }$C, the predictions made for the samples conditioned at relative humidity 35%, do not match the experimental values, see Figure 25. We can only speculate about this mismatch.

### 4.5. Structures-Properties Relations for Modelling

**Figure 26:**
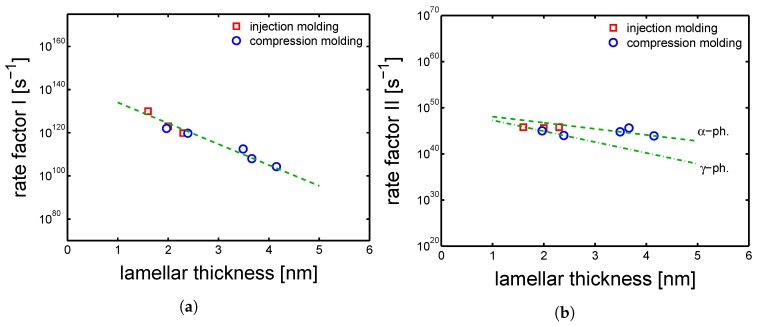
(**a**) Rate factor I and (**b**) rate factor II as functions of lamellar thickness. Lines are guides to the eye.

As mentioned in Section 2, a previous study [9] on samples crystallized quiescently, a relation between the lamellar thickness and the rate factors (${\dot{\epsilon }}_{0,I,II}$) was proposed. In Figure 26a,b, the rate factors I and II defined for the samples processed by injection molding are plotted as functions of lamellar thickness together with the rate factors obtained for compression molding. In the case of the rate factor I, the injection molding markers are in good agreement with the trend obtained in the case of compression molding, see Figure 26a. Plotting the ${\dot{\epsilon }}_{0,II}$ obtained for injection molding samples as a function of lamellar thickness, values are in line with trend found for compression molding, see Figure 26b. However, because of the mixture of α and γ crystals present in the sample molded by injection molding, the trends might results not perfectly in line with the polymorph division (see dashed lines in Figure 26b).

## 5. Conclusions

In this study, an industrial injection molding machine was used to produce polyamide 6 samples with different crystallographic properties. The mold temperature was varied in a range from 35 to 160 ${}^{\circ }$C. A clear influence of mold temperature on the crystallization was detected. In the investigated range of temperatures, different crystallinity values, lamellar thicknesses and crystal polymorphs were obtained. Moreover, also the effect of shear flow and pressure during crystallization were studied by dilatometry (PVT). This led to the conclusion that the well known shift of the crystallization to higher temperature is dominant, while shear flow has a minor effect on crystallization kinetics. The effect of pressure during injection molding was particularly highlighted by a deconvolution analysis performed on WAXD patterns obtained for samples made with different mold temperatures. To confirm this finding, the deconvolution analysis performed on samples obtained by injection molding was compared with samples obtained by compression molding (in which pressure and shear flow effects are negligible). It was found that in the case of injection molding, even at a mold temperature of 160 ${}^{\circ }$C, a fully α-phase sample was obtained; while in the case of compression molding at the same temperature, a mixture of α and γ was obtained. This effect was ascribed to the high pressure present during cooling in the case of injection molding. By azimuthal integration, the presence of molecular orientation was measured. Orientation was found but, surprisingly, the mechanical properties did not show an influence of the orientation. The mechanical properties were tested by uniaxial tensile tests and uniaxial creep tests at several temperature in dry condition and at room temperature with varying relative humidity. The Eyring’s flow model, modified with the apparent temperature and combined with the concept of critical strain, were employed in order to describe the results obtained by tensile and creep tests. This was partially successful. In the case of samples molded at 160 ${}^{\circ }$C and 130 ${}^{\circ }$C, the model was successfully applied by the use of parameters previously determined as characteristic in the case of α-phase and γ-mesophase, respectively. In the case of mold temperature 85 ${}^{\circ }$C, it was hypothesized that the inhomogeneous morphology (intrinsically induced by the molding technique) led to an excessive contribution of the amorphous layer (skin layer). Finally, in the case of mold temperature 35 ${}^{\circ }$C, in which completely amorphous samples were obtained, a description was not achieved due to the continuous development of the micro-structure. 

## Figures and Tables

**Table 1:** Ree-Eyring parameters defined for α-phase.

	$V^{*}$ $\left[m^{3}\right]$	ΔU $\left[{J\;\mathrm{mol}}^{-1}\right]$
*I*	9 $\times \;10^{-27}$	1 $\times \;10^{6}$
*II*	6 $\times \;10^{-27}$	3.2 $\times \;10^{5}$

**Table 2:** Ree-Eyring parameters defined for γ-mesophase.

	$V^{*}$ $\left[m^{3}\right]$	ΔU $\left[{J\;\mathrm{mol}}^{-1}\right]$
*I*	9 $\times \;10^{-27}$	1 $\times \;10^{6}$
*II*	1.9 $\times \;10^{-27}$	3 $\times \;10^{5}$

**Table 3:** Ree-Eyring parameters: rate factors for samples molded at 160 ${}^{\circ }$C.

	$\dot{\epsilon_{0}}$ $\left[s^{-1}\right]$
*I*	7 $\times \;10^{119}$
*II*	6 $\times \;10^{45}$

**Table 4:** Ree-Eyring parameters: rate factors for samples molded at 130 ${}^{\circ }$C.

	$\dot{\epsilon_{0}}$ $\left[s^{-1}\right]$
*I*	1 $\times \;10^{123}$
*II*	4 $\times \;10^{45}$

**Table 5:** Ree-Eyring parameters: rate factors for samples molded at 85 ${}^{\circ }$C.

	$\dot{\epsilon_{0}}$ $\left[s^{-1}\right]$
*I*	1 $\times \;10^{130}$
*II*	6 $\times \;10^{45}$

**Table 6:** Glass transition temperature [${}^{\circ }$C].

Sample	Dry	35 RH%	50 RH%	75 RH%
mold 160 ${}^{\circ }$C	68	41	26	10
mold 130 ${}^{\circ }$C	73	39	25	11
mold 85 ${}^{\circ }$C	71	38	25	10
mold 35 ${}^{\circ }$C	56	45	26	15
